# Thermal Stability and Thermoelectric Properties of NaZnSb

**DOI:** 10.3390/ma12010048

**Published:** 2018-12-24

**Authors:** Volodymyr Gvozdetskyi, Bryan Owens-Baird, Sangki Hong, Julia V. Zaikina

**Affiliations:** 1 Department of Chemistry, Iowa State University, Ames, IA 50011, USA; volodya@iastate.edu (V.G.); bowens@iastate.edu (B.O.-B.); skhong@iastate.edu (S.H.); 2 Ames Laboratory, U.S. Department of Energy, Ames, IA 50011, USA

**Keywords:** antimonide, thermoelectric, alkali metal, hydride, zinc, crystal structure, Zintl

## Abstract

A layered Zintl antimonide NaZnSb (PbClF or Cu_2_Sb structure type; *P*4/*nmm*) was synthesized using the reactive sodium hydride NaH precursor. This method provides comprehensive compositional control and facilitates the fast preparation of high-purity samples in large quantities. NaZnSb is highly reactive to humidity/air and hydrolyzes to NaOH, ZnO, and Sb in aerobic conditions. On the other hand, NaZnSb is thermally stable up to 873 K in vacuum, as no structural changes were observed from high-temperature synchrotron powder X-ray diffraction data in the 300–873 K temperature range. The unit cell expansion upon heating is isotropic; however, interatomic distance elongation is not isotropic, consistent with the layered structure. Low- and high-temperature thermoelectric properties were measured on pellets densified by spark plasma sintering. The resistivity of NaZnSb ranges from 11 mΩ∙cm to 31 mΩ∙cm within the 2–676 K range, consistent with heavily doped semiconductor behavior, with a narrow band gap of 0.23 eV. NaZnSb has a large positive Seebeck coefficient (244 μV∙K^−1^ at 476 K), leading to the maximum of *zT* of 0.23 at 675 K. The measured thermoelectric properties are in good agreement with those predicted by theoretical calculations.

## 1. Introduction

Zintl phases represent a special class of intermetallic compounds [1,2]. Classical Zintl compounds consist of cationic entities (electropositive alkali or alkali earth metals) and anionic fragments composed of *p*-elements from Group 13 to 16. The *p*-elements within the anionic fragments satisfy an octet through formation of covalent bonds and lone pair localization. Such bonding patterns often lead to complex structures and electron-balanced compositions. In recent decades, the Zintl phase family has considerably expanded and now includes examples containing rare-earth elements and transition metals [3,4,5,6].

Complex structures of Zintl phases with large unit cells, atomic sites with mixed occupancy, and the possibility for charge carrier adjustments through doping are the key factors responsible for their observed high thermoelectric efficiency [3,4,5], i.e., their ability to convert heat into electric energy and vice versa. Zintl antimonides [4] stand out as a promising class of thermoelectric materials, often exhibiting low thermal conductivities and amenability for doping. For instance, Yb_14_MnSb_11_ has been shown to be a superb *p*-type thermoelectric material for high-temperature applications [6]; this material is currently being tested at the Jet Propulsion Laboratory (JPL) for the next generation of radioisotope thermoelectric generators (RTGs) [7]. Several other Zintl antimonides exhibit good thermoelectric performance at high temperatures: Ca_9_Zn_4+x_Sb_9_, EuZn_2_Sb_2_, Yb_9_Mn_4.2_Sb_9_, YbCd_2_Sb_2_, and *Ae*Zn_2_Sb_2_ (*Ae* = Ca, Sr, Ba, Eu, or Yb) to name just a few [4]. Beyond the intrinsic material performance, the evaluation of the thermal stability of these compounds is important for high-temperature applications. While Yb_14_MnSb_11_ shows remarkable stability [6,7], recent studies of the SrZn_2_Sb_2_ Zintl antimonide [8] have shown that it is not suitable for high-temperature applications due to structural instability at elevated temperatures.

We are interested in ternary antimonides containing alkali metals. Using the unconventional hydride synthetic route [9,10], K_8−x_Zn_18+3x_Sb_16_ was recently discovered [11]. Here, we report the thermoelectric properties and thermal stability of NaZnSb, studied using a comprehensive set of methods including high-temperature powder X-ray diffraction and differential scanning calorimetry. The thermoelectric properties of NaZnSb are then compared with those predicted from theoretical calculations.

## 2. Materials and Methods

### 2.1. Synthesis

Chemicals: Zinc powder (Zn, AlfaAesar, 99.996%), antimony lump (Sb, AlfaAesar, 99.9999%), sodium metal (Na, AlfaAesar, 99.95%), and sodium hydride NaH (Sigma-Aldrich, 95%) were used as received. All manipulations were carried out in a glove box under a dry argon atmosphere, *p*(O_2_) < 1 ppm, *p*(H_2_O) < 1 ppm.

Hydride route: Powders of the starting materials were weighed in a NaH/Zn/Sb = 1.03:1:1 molar ratio (total mass: 0.7 g) and loaded into a polypropylene grinding set with a methacrylate grinding ball. The vial was sealed in two polyethylene bags under an argon atmosphere and ball milled using a SPEX mixer/mill 8000M for 6 min. Afterwards, inside an argon-filled glove box, the freshly prepared fine powders were loaded into tantalum containers and sealed shut with an arc welder. The Ta containers were then placed into silica reactors equipped with Swagelok safety check valves to prevent over-pressurizing of the reactors due to hydrogen gas release during the heat treatment. The silica reactors were evacuated down to 4 × 10^−5^ bar pressure, placed into a high-temperature furnace, and slowly (2 K∙min^−1^) heated from room temperature to 873 K. The ampoules were held at that temperature for 16 hours and cooled to room temperature by switching the furnace off.

Synthesis from elements: For single crystal growth, a synthesis from elements was employed. Inside the glove box, elemental Na, Zn, and Sb in a 1.1:1:1 molar ratio (total mass: 0.3 g) were loaded into carbonized quartz ampoules, evacuated down to 4 × 10^−5^ bar pressure, placed into a high-temperature furnace, and heated (1.83 K∙min^−1^) from room temperature to 823 K. The ampoules were held at that temperature for 24 hours and cooled to room temperature by switching the furnace off.

### 2.2. Characterization

Powder X-ray Diffraction (PXRD): The purity of polycrystalline samples was checked by means of X-ray powder diffraction using a Rigaku MiniFlex600 powder diffractometer (Rigaku Corporation, Tokio, Japan) with Cu Kα radiation (*λ* = 1.54051 Å) and a Ni Kβ filter. Data were collected on an air-sensitive zero-background plate holder at room temperature. Phase analysis was performed using the PDF-2 database incorporated into the PDXL program software package [12].

Single-Crystal X-ray Diffraction (SC XRD): Single crystal data were collected using a Bruker D8 VENTURE diffractometer ((Bruker Corporation, Billerica, MA, USA) (Photon CMOS detector, Mo-IμS microsource, and Oxford Cryosystem 800 low-temperature device) at 173 K. Data integration, absorption correction, and unit cell determination were performed by the APEX 3 software [13]. The crystal structure solution was performed in the centrosymmetric space group *P*4/*nmm* (no. 129). The positions of Na, Zn, and Sb were obtained by direct methods with SHELXS-2017 [14]. Subsequently, the structure was refined using SHELXL-2017 () [14] (full-matrix least-squares on *F_o_*^2^). The site occupancies for all atomic positions were refined separately and in succession, showing no deviation from unity within estimated standard deviations (e.s.d.). The final refinement with anisotropic atomic displacement parameters for all atoms converged to *R*_1_ = 0.02, leading to the composition NaZnSb. Details of the data collection and refinement are summarized in Tables S1 and S2.

Spark Plasma Sintering (SPS): Consolidation of the NaZnSb powders was performed via Spark Plasma Sintering (SPS). In an argon-filled glovebox, the powdered samples were loaded into a small graphite die (inner diameter: 5 mm) between several circles of graphite foil and pressed with tungsten carbide WC plungers. The assembled die was further inserted into a larger outer graphite die with graphite plungers (inner diameter 20 mm), taken out of the glovebox, and quickly set up for sintering in the SPS unit Dr. Sinter Lab Jr. SPS-211Lx (FUJI ELECTRONIC IND. CO., LTD., Fujimi, Japan). For SPS of 12.7 mm pellets, a larger graphite die and graphite plungers were used. The samples were sintered by slow heating to 623 K over a period of 5 min, with subsequent further heating to 673 K in 3 minutes, and dwelling for 10 minutes under a uniaxial pressure of 90 MPa. Afterwards, the pressure was released, and the sintered pellets were allowed to cool to room temperature without the application of pressure. Finally, the pellets were pressed out of the graphite dies and polished to remove traces of the graphite foil. The geometrical density of the pellets was 100% of the theoretical X-ray density.

Differential scanning calorimetry and thermogravimetric analysis (DSC-TGA): To evaluate the thermal stability of NaZnSb, a Differential Scanning Calorimetry (DSC) measurement was performed using a Netzsch 404 F3 Pegasus Differential Scanning Calorimeter (Netzsch Group, Selb, Germany). A powdered sample (mass: 50.00 mg) was loaded into a silica ampoule which was further sealed under vacuum. The sample was heated to 973 K and cooled down to room temperature with a 10 K∙min^−1^ rate. In a separate run, the thermal stability of a piece of a pellet densified by SPS (mass: 11.00 mg) and a powdered sample (9.80 mg) was checked by DSC/TGA measurement in an alumina (Al_2_O_3_) pan with a lid using a Netzsch STA449 F1 Jupiter. Samples were heated to 890 K and cooled down to 360 K with a 10 K∙min^−1^ rate in a flow of argon.

Elemental Analysis: Semiquantitative elemental analysis was performed with energy-dispersive X-ray spectroscopy (EDXS) using a FEI Quanta-250 field emission scanning electron microscope (Thermo Scientific^TM^, Walthalm, MA, USA) equipped with an Oxford X-Max 80 detector and an Oxford Aztec energy-dispersive X-ray analysis system. The polished pieces of SPS sintered pellets were mounted in an argon atmosphere onto a home-built holder designed for air-sensitive samples using a double-sided carbon tape. Samples were oriented with a flat face perpendicular to the beam and analyzed using a 15 keV accelerating voltage and an accumulation time exceeding 60s.

High-temperature synchrotron powder X-ray Diffraction (HT PXRD): High-temperature X-ray diffraction data were collected at the synchrotron beamline 17-BM at the Advanced Photon Source (APS) at Argonne National Lab (ANL), λ = 0.24130 Å, for a sample of NaZnSb synthesized via the hydride route. A powdered sample of NaZnSb, prepared from hydrides, was filled into a 0.7 mm outer diameter thick-wall (0.1 mm) silica capillary and sealed under vacuum. The capillary was mounted into a secondary shield capillary located on a sample stage equipped with two resistive micro-heaters and a thermocouple set as close as possible to the measurement area. The further details of experimental setup can be found elsewhere [15]. Data were collected upon heating and cooling in the temperature range 298 K–868 K–298 K with a heating and cooling rate of 10 K∙min^−1^.

Diffraction patterns were analyzed by the Rietveld refinement method using the GSAS II software package [16]. The profile parameters, background parameters, and cell parameters were refined first. The background was fitted using a shifted 14-order Chebyshev polynomial function, and a pseudo-Voigt function was applied to generate the peak profile shape. The *z*-coordinates of Na and Sb occupying two 4*d* sites with (¼; ¼; *z*) coordinates (*P*4/*nmm*, no. 129) were refined.

Thermoelectric properties measurement: The low-temperature transport properties of the pellet (∅: 5 mm) prepared by SPS were measured in the temperature range of 2–300 K using the commercial multipurpose Physical Properties Measurement System Evercool II (PPMS, Quantum Design Inc., San Diego, CA, USA). The Seebeck thermopower and thermal conductivity were measured using the Thermal Transport Option (TTO) in a two-probe configuration. The high-temperature transport properties for the SPS-prepared pellet (∅: 12.7 mm) were determined using Netzsch LFA467 HT Hyper Flash and Netzsch SBA 458 Nemesis instruments in the temperature range 300−673 K. For the thermal conductivity measurements, a standard sample of Pyroceram 9606 was used as the reference for estimating heat capacity. The thermal conductivity was calculated from the relationship *κ* = *D* × *ρ* × *C_P_* (*D*, thermal diffusivity; *ρ*, density; *C_P_*, heat capacity). The high-temperature electrical resistivity and Seebeck coefficient were measure using a Netzsch 458 Nemesis in a four-probe geometry of the round pellet. After the high-temperature transport property measurements were completed, the same pellet (∅: 12.7 mm) was cut into a bar-shaped sample of suitable sizes to perform low-temperature electrical conductivity measurements using the alternating current (AC) transport option in the PPMS and a four-probe geometry with 50 μm platinum wires attached using silver paste. The combined measurement uncertainty in the thermoelectric figure of merit is generally assumed to be ∼20% [17].

## 3. Results and Discussion

### 3.1. Crystal Structure and Electron Count

**Figure 1:**
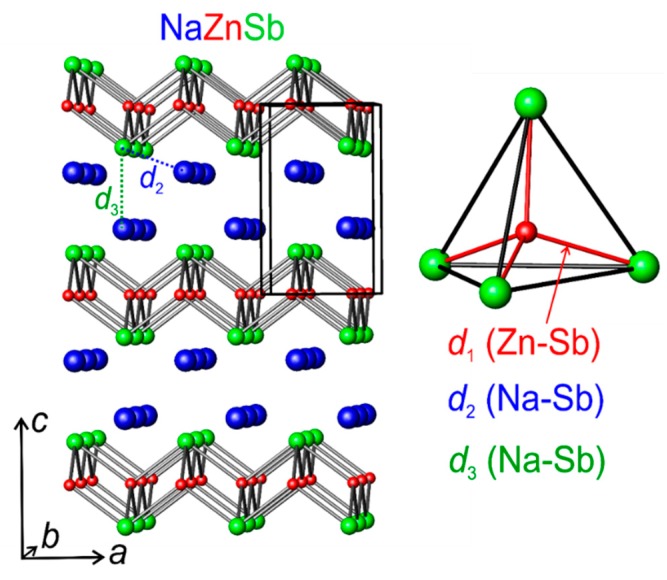
Crystal structure of NaZnSb. The tetrahedral coordination of Zn by four Sb atoms is shown on the right. The selected interatomic distances are color coded. Atom color coding: Na, blue; Zn, red; Sb, green.

NaZnSb was first reported by Schuster et al. back in 1976 [18]. It is a layered zinc pnictide (Figure 1), isostructural to NaFeAs and LiFeAs 111-superconductors (PbClF or Cu_2_Sb structure type, *P*4/*nmm*). The NaZnSb structure was determined from SC XRD data (Table 1 and Table 2), and it is consistent with the previous report [18]. The structure is composed of three crystallographic positions, one for each element within the structure: Na (*2c*), Zn (*2a*), and Sb (*2c*) (Table 2). NaZnSb has a layered structure composed of alternating bilayers of Na^+^ cations and anionic [ZnSb]^−^ slabs with Na–Sb distances of 3.27 Å. Within the [ZnSb] layer, Zn atoms are tetrahedrally coordinated by four Sb atoms, and Sb has the same coordination with Zn–Sb distances of 2.76 Å and Zn–Zn distances of 3.13 Å. The ZnSb_4_ tetrahedra are slightly distorted, with two bond angles of 106° and 110°. The refinement of occupancy of all atomic sites, particularly Na, did not reveal any deviations from unity within 3 e.s.d.

The charge assignment using traditional oxidation states can be done by considering that the electrons from Na^+^ and Zn^2+^ cations are fully donated to anionic Sb^3−^. Using the Zintl counting methodology, a charge balanced composition can be achieved in Na^+^Zn^2−^Sb^+^, considering that 4-bonded Sb with 5 valence electrons has one excess electron. In turn, 4-bonded Zn, with 2 valence electrons, requires 2 more electrons to achieve an octet. Nonetheless, the charge balance for both electron counting schemes is suggestive of semiconducting behavior. The calculated band structure depends on the method used: NaZnSb is predicted to be metallic using the full potential linear augmented plane wave (FPLAPW) method within local density approximation (LDA), generalized gradient approximation (GGA), or Engel–Vosko (EV-GGA) approximation [19,20]. However, NaZnSb is predicted to have a narrow direct band gap of 0.25 eV when calculations are performed using the FPLAPW method with the modified Becke–Johnson potential [21].

### 3.2. Synthesis

Synthesis from elements led to a single-phase sample; however, mass scale-up proved to be difficult, as increased Na resulted in side reactions and impurity phases. We have shown that by using alkali metal hydrides, homogeneous mixing and control over the local concentration of alkali metal can be achieved, leading to substantially shorter reaction times and higher yields of the target phase [9,10,11]; while for the traditional synthesis with elemental alkali metals as the starting materials, long reactions at high temperatures, prereactions, and subsequent grindings of the samples are required to facilitate diffusion and achieve impurity-free samples. The ductility of the elemental alkali metals prevents their thorough mixing with other starting materials, often leading to alkali-metal-rich phases. By using the reactive alkali metal hydride NaH precursor as a source of sodium, the reaction is completed within a span of 12 hours. This synthesis can also be easily scaled up to prepare a sufficient amount of sample to further study its high-temperature transport properties.

### 3.3. Air Sensitivity and Thermal Stability

The stability of ternary zinc antimonides in air correlates to some extent with their crystal structure. For instance, Cs_8_Zn_18_Sb_28_ clathrate type-I [22], in which Cs^+^ cations are trapped inside large polyhedral cages of Zn and Sb, is air- and water-stable. Recently discovered, K_8−x_Zn_18+3x_Sb_16_ [11] has open channels in the Zn–Sb framework filled by alternating K^+^ cations and Zn_3_ triangles. The alternating Zn_3_ triangles block the “diffusion” of the K^+^ from the structure and are believed to be a reason for the observed stability of K_8−x_Zn_18+3x_Sb_16_ in air. In turn, the layered structures *A*ZnSb (*A* = Li, Na, K) are air- and water-sensitive, suggestive of the mobility of alkali metal within the layers leading to the phase degradation. The samples of NaZnSb prepared from either elements or hydrides are found to be air-sensitive. When exposed to air, NaZnSb degrades, as zinc oxide and elemental antimony were detected by powder XRD as products of the oxidation.

Evaluation of the thermal stability of zinc antimonides is important if high-temperature thermoelectric properties are to be accessed. For instance, SrZn_2_Sb_2_ was previously reported to exhibit a maximum *zT* of 0.35 at 723 K [23]. This antimonide was further studied for its thermal stability in air, argon, and vacuum atmospheres, for both powdered and pelletized samples [8]. It should be noted that the studied samples contained only up to 90% of the target SrZn_2_Sb_2_ phase. The high-temperature synchrotron powder X-ray diffraction experiments of SrZn_2_Sb_2_ in air revealed its decomposition at 500 K. DSC measurement of the powdered sample performed in an argon flow indicated kinetically slow decomposition at 882 K, accompanied by a weight loss associated with zinc evaporation. During the SPS sintering, the authors also observed degradation of the sample at 723 K. Finally, the heat treatment of cold-pressed pellets of SrZn_2_Sb_2_ for five days at 850 K in vacuum or argon revealed a complete decomposition of the phase. Thus, it was concluded that SrZn_2_Sb_2_ is intrinsically unstable in the intermediate- to high-temperature region, regardless of the atmosphere and compaction degree, making it highly unsuitable for thermoelectric applications.

We have studied the thermal stability of NaZnSb by means of (1) high-temperature synchrotron powder XRD (HT PXRD) in a capillary sealed under vacuum; (2) DSC of a powdered sample in a silica ampoule sealed under vacuum; and (3) DSC/TGA of an SPS pellet and powdered sample in an open alumina pan crucible under argon flow.

**Figure 2:**
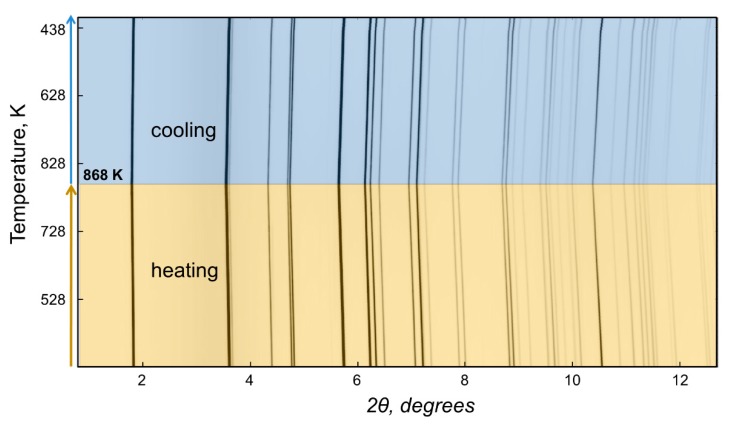
High-temperature synchrotron powder X-ray diffraction patterns for the NaZnSb powdered sample sealed in a silica capillary under vacuum. The “waterfall” diagram shows the evolution of XRD patterns with the concomitant change upon heating from room temperature to 868 K followed up by cooling back to room temperature. The intensity of the diffraction peaks is represented by the color intensity. Heating/cooling regions are highlighted in yellow/blue. All of the diffraction peaks can be ascribed to NaZnSb.

The HT PXRD of the NaZnSb sample prepared from NaH revealed no decomposition of the target phase (Figure 2) when heated up to 868 K in the evacuated and flame-sealed silica capillary. The unit cell parameters obtained from Rietveld refinement of the HT PXRD data linearly increase upon heating with no hysteresis for heating/cooling cycling. The coefficients of thermal expansion (CTE) obtained from linear fits of the data were calculated according to the following equations:

(1) $$\mathrm{CTE}\;(a)\;=\;\frac{da}{dT}\times \frac{1}{a}\;=\;30.1(2)\;\times \;10^{-6}\;K^{-1},$$

(2) $$\mathrm{CTE}\;(c)\;=\;\frac{dc}{dT}\times \frac{1}{c}\;=\;33.9(1)\;\times \;10^{-6}\;K^{-1},$$

(3) $$\mathrm{CTE}\;(V)\;=\;\frac{dV}{dT}\times \frac{1}{V}\;=\;95.8(4)\;\times \;10^{-6}\;K^{-1}.$$

**Figure 3:**
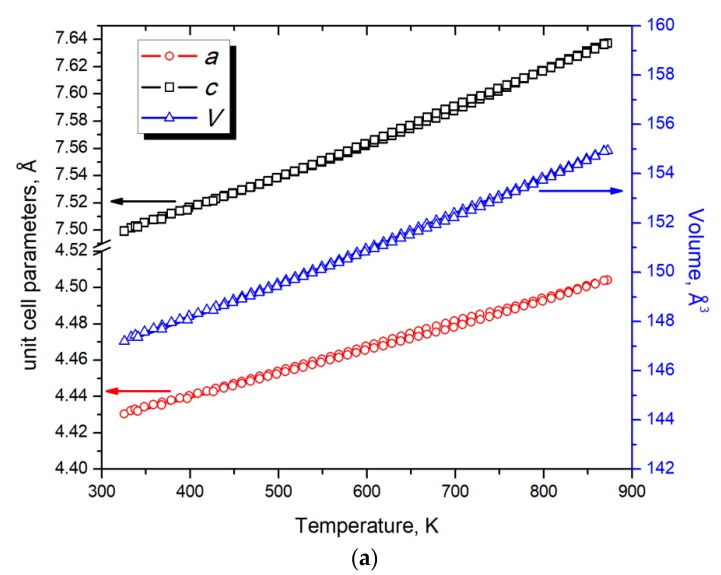

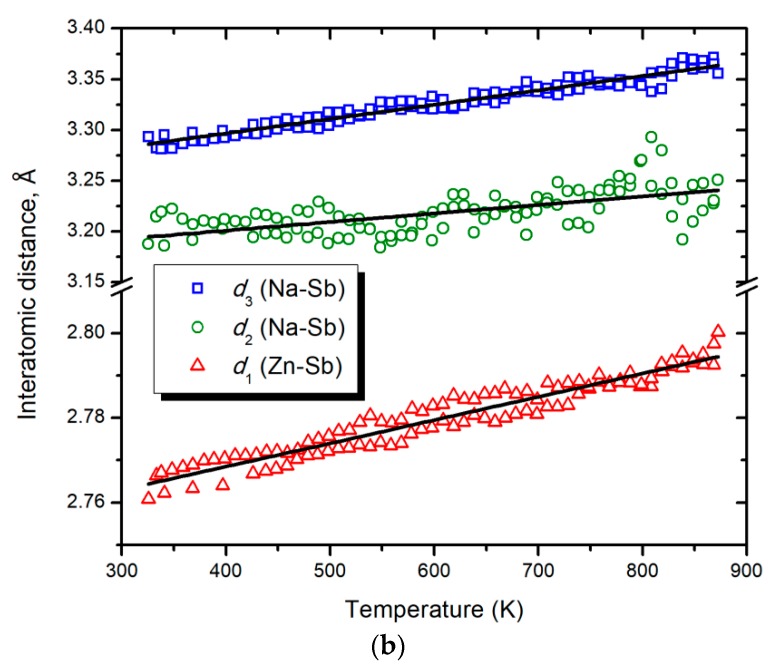
(**a**) Temperature dependence of unit cell parameters (*a* and *c*) and unit cell volume (*V*) for NaZnSb; (**b**) Temperature dependence of the selected interatomic distances in the structure of NaZnSb: d_1_(Zn–Sb), d_2_(Na–Sb), and d_3_(Na–Sb) (see Figure 1). The black lines correspond to the linear fit of the data.

The unit cell expansion upon heating is isotropic, and both the *a* and *c* parameters of the tetragonal unit cell increase at nominally the same rate. However, the interatomic distances increase at a different rate with increasing temperature. The Zn–Sb distances (d_1_) and Na–Sb distance (d_2_) are affected by heating to a lesser extent compared to the Na–Sb distance along the *c* axis (d_3_) (Figure 1 and Figure 3):

(4) $$\mathrm{CTE}\;(d_{1})\;=\;\frac{dd_{1}}{dT}\times \frac{1}{d_{1}}\;=\;19.9(4)\;\times \;10^{-6}\;K^{-1},$$

(5) $$\mathrm{CTE}\;(d_{2})\;=\;\frac{dd_{2}}{dT}\times \frac{1}{d_{2}}\;=\;26(3)\;\times \;10^{-6}\;K^{-1},$$

(6) $$\mathrm{CTE}\;(d_{3})\;=\;\frac{dd_{3}}{dT}\times \frac{1}{d_{3}}\;=\;43(1)\;\times \;10^{-6}\;K^{-1}.$$

This behavior can be attributed to some degree of anisotropy of the layered NaZnSb structure.

**Figure 4:**
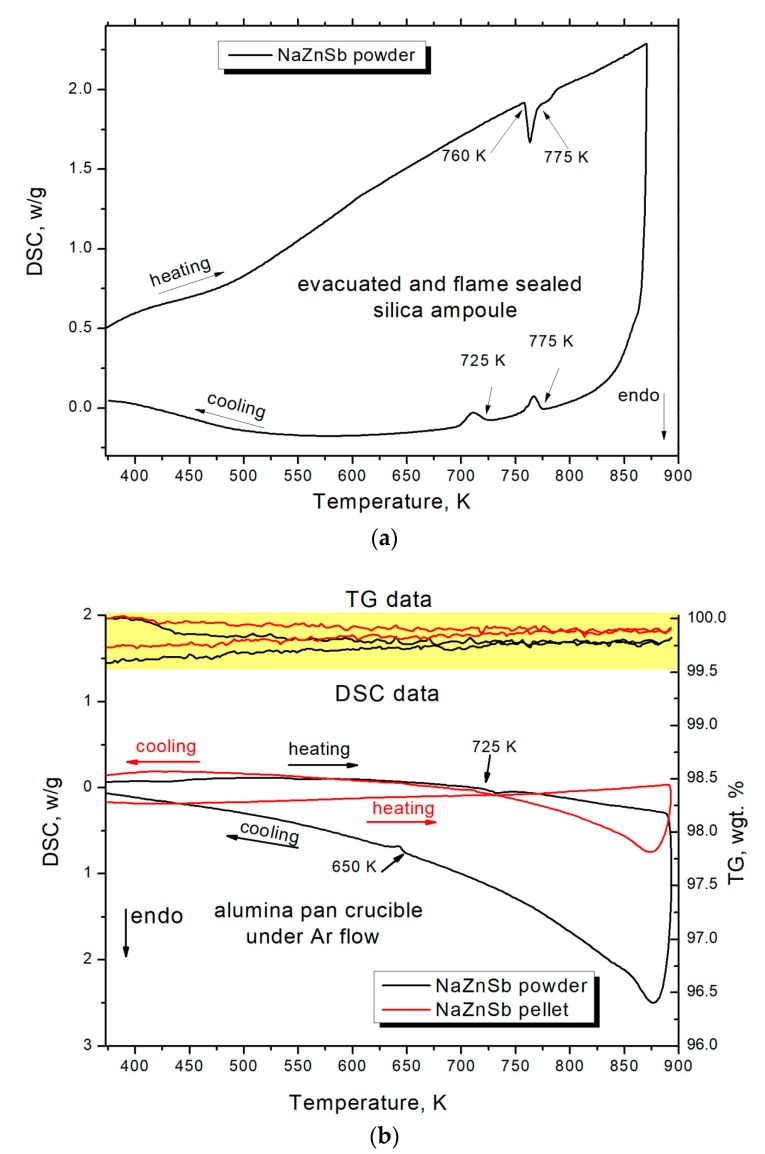
DSC data of the NaZnSb sample obtained for a powdered sample of NaZnSb in an evacuated and flame-sealed silica ampoule (**a**) and combined DSC-TG data for the powdered (black) and SPS sintered (red) samples of NaZnSb measured in an open alumina pan crucible in Ar flow (**b**). Samples were heated from room temperature to 873 K and cooled back to room temperature at a rate of 10 K∙min^−1^. Highlighted in yellow is TG data with the TG axis, wt % to the right.

**Figure 5:**
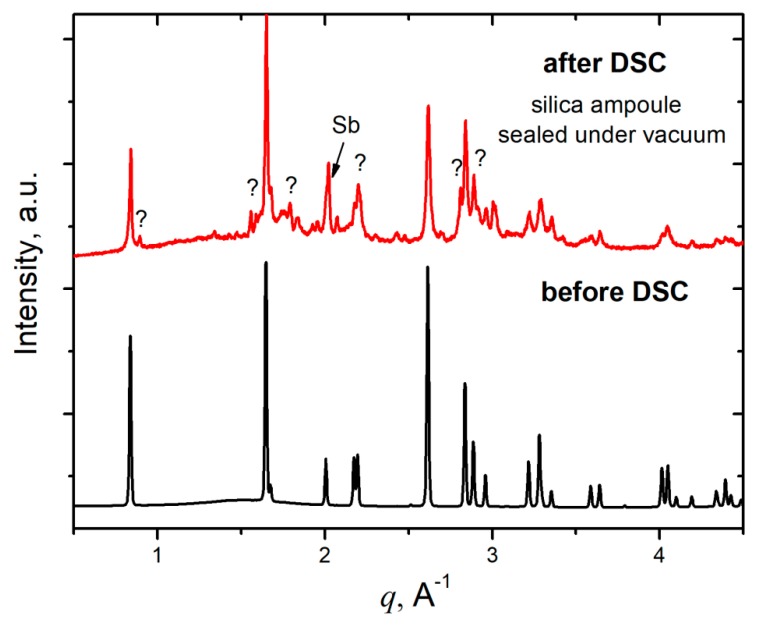
Comparison X-ray powder diffraction patterns of NaZnSb before (black) and after (red) DSC measurement. Data were collected using laboratory PXRD (after) and synchrotron PXRD (before). Before the DSC measurement, the pattern corresponds to the calculated pattern of NaZnSb phase; thus, no impurities are present. Traces of Sb were detected after DSC measurement in an evacuated and flame sealed silica capillary together with a plethora of new peaks emerging in the powder diffraction pattern, indicating the formation of unknown compound(s).

The DSC measurement in an evacuated and flame-sealed silica ampoule revealed the endothermic processes at 760 K and 775 K during heating followed by two exothermic signals at 775 K and 725 K upon cooling (Figure 4a). The PXRD of the NaZnSb sample after the DSC run is indicative of partial decomposition of the initial NaZnSb phase. Additional peaks in the PXRD pattern (Figure 5) correspond to elemental antimony and a new Na_1−x_ZnSb ternary phase, whose structure and properties are currently being investigated. The difference between HT synchrotron powder XRD data and DSC measurement can be a result of the side reaction between Na leached from NaZnSb and silica upon heating [24]. The ratio between the surface area of the silica DSC ampoule and sample is greater in the DSC experiment, promoting the side reaction of Na with SiO_2_. Additionally, flame sealing of a short DSC ampoule may result in partial decomposition of NaZnSb.

To further study this, DSC experiments in an alumina pan crucible of the SPS-densified NaZnSb pellet and NaZnSb powders were performed in a flow of argon. The minor weight loss of ~0.5 wt % might be a result of Na/Zn subtle evaporation, although the mass loss is not coincident with a DSC peak. For comparison, in the case of SrZn_2_Sb_2_, the weight loss is considerable: ~15 wt % at 882 K with a very broad DSC peak at 882 K [8]. The PXRD pattern of the sample after the DSC-TG run corresponds to a parent NaZnSb compound with traces of an elemental Sb impurity, in line with negligible mass loss. The difference in the DSC signal in the case of pellet and powder is due to different contact areas between the bottom of the crucible and the sample. It should be noted that the temperatures of the DSC peaks in all cases are lower than the melting points of Zn–Sb binary compounds (>808 K) and elemental Sb (903 K) [25].

Furthermore, heat treatment of NaZnSb in an evacuated and flame-sealed silica ampoule (*m*(NaZnSb) = 0.05 g; 5 mm diameter, 10 cm length) at 750 K for 4 hours results in partial decomposition of NaZnSb, with traces of elemental Sb detected by PXRD. This could be due to reactions with the silica vessel and negligible evaporation of Na and Zn. A short heat treatment (6 min) in the longer ampoule (36 cm length) at an increased temperature of 1173 K resulted in a complete decomposition of NaZnSb, with NaSb and Sb products and a deposition of Zn metal in the cold part of the tube.

Thus, HT PXRD and DSC-TG data suggest that NaZnSb is stable up to 870 K in vacuum or inert atmosphere, although its stability can be affected by the side reaction of Na with the crucible material, suggesting that Na^+^ cations are labile in NaZnSb. The different stability of NaZnSb upon heating in an evacuated and flame-sealed silica capillary, DSC ampoule, or longer ampoules suggests that another factor influencing the NaZnSb thermal stability is the partial pressure of Na/Zn. This data also emphasizes the importance of considering partial pressure as well as the crucible material in accessing the thermal stability of alkali metal zinc pnictides via DSC and HT XRD.

### 3.4. Thermoelectric Properties

The temperature-dependent thermoelectric properties were measured for the SPS sintered pellets of NaZnSb (compactness of 100%) in the 2–675 K temperature range. Scanning electron microscopy (SEM) and energy-dispersive X-ray (EDX) analysis indicate a homogeneous elemental distribution and a uniform microstructure (Figure S1). The averaged composition determined by EDX is Na_1.3(1)_Zn_1.0(1)_Sb_1.0(1)_. The Na content is overestimated due to the overlap between Na Kα and Zn Lα leading to difficulties in deconvoluting the Na and Zn characteristic lines in the spectrum. Additionally, the considerable air sensitivity of NaZnSb may result in a partially oxidized surface enriched in sodium oxide, Na_2_O.

The efficiency of a thermoelectric material can be assessed by a calculation of its dimensionless figure of merit: *zT* = *S*^2^∙*T*/*ρ*∙*κ*, where *S* is the Seebeck coefficient or thermopower, *T* is an absolute temperature, *ρ* is the electrical resistivity, and *κ* is the thermal conductivity. The electronic and thermal transport properties are interdependent, making the optimization of *zT* for a material difficult. An optimized charge carrier concentration (high *S* and low *ρ*) needs to be coupled with slow transport of heat-carrying phonons (low *κ*), leading to the enhancement of *zT*.

**Figure 6:**
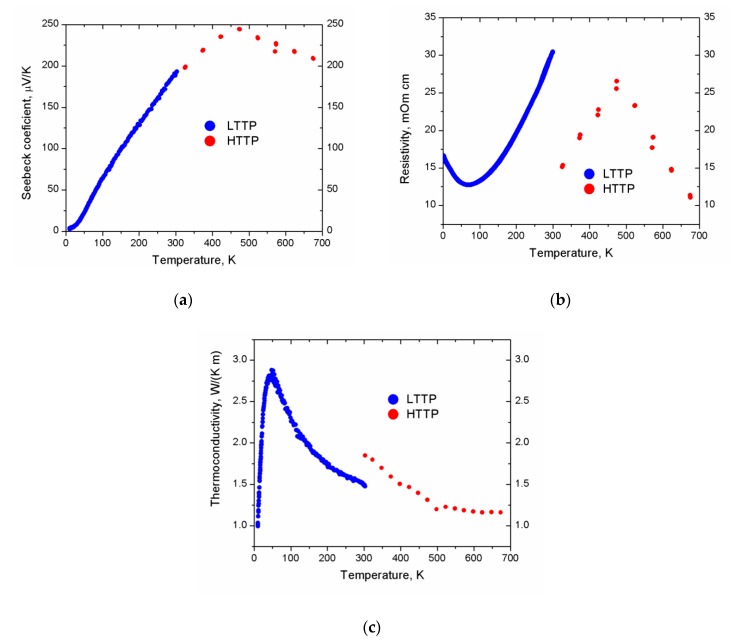
Temperature dependence of transport for NaZnSb: (**a**) Seebeck coefficient, (**b**) electrical resistivity, (**c**) total thermal conductivity (the estimated electronic thermal conductivity contribution is negligible, less than 2%, and is not shown). The low-temperature thermoelectric properties (LTTP) data are shown in blue, while the high-temperature thermoelectric properties (HTTP) data are shown in red. The discontinuity between LLTP and HTTP is due to the different instruments used for the measurement.

The high- and low-temperature thermoelectric properties for two pellets (5 mm diameter and 12.7 mm diameter, respectively) of NaZnSb prepared via the hydride route are shown in Figure 6. The discontinuity of the data seen in the measured transport properties at 300 K is due to the change of instruments and is common.

The electrical resistivity of NaZnSb (Figure 6) is temperature dependent, ranging from 11 mΩ∙cm to 31 mΩ∙cm within the 2–676 K temperature range. There are two changes of the slope for the resistivity data: from heavily doped semiconductor-like to metallic at ~67 K and back to semiconductor-like at 476 K. The material has a large positive Seebeck coefficient, indicative of *p*-type conduction and holes as the main charge carriers. Seebeck coefficient steadily increases with temperature up to 244 μV∙K^−1^ at 476 K, with a further decrease down to 208 μV∙K^−1^ at 676 K. The thermal conductivity of NaZnSb increases with temperature up to 50 K followed by a steady decrease to 1.12 W∙m^−1^∙K^−1^ at 676 K, behavior typical for highly crystalline solids.

The temperature dependence of the electrical resistivity suggests heavily doped semiconducting behavior with a very narrow band gap. This is consistent with the previously reported [19,20,21] band structure calculations, which depending on the calculation method used, predict NaZnSb to be either metallic or to have a narrow band gap of 0.25 eV (vide supra). The change in the slope of the resistivity at 67 K can be either attributed to a low-temperature semiconductor-to-metal transition or can be accounted for by the partial oxidation of the sample. The sample was briefly exposed to air before low-temperature measurements, and the low-temperature resistivity measurement was the last measurement performed. At 476 K there is a change of slope for resistivity, Seebeck coefficient, and thermal conductivity. This observation cannot be due to a structural transition, as the high-temperature synchrotron X-ray data revealed no structural changes up to 868 K and the linear coefficients of thermal expansion (CTE) of the unit cell parameters and interatomic distances. In turn, this can be attributed to a bipolar effect, e.g., the thermal excitation of minority charge carriers across the band gap (electrons in the case of *p*-type NaZnSb) leading to the reduction of thermopower, thus resulting in a peak at *S*(*T*) [26,27]. The band gap of NaZnSb can then be calculated from the maximum thermopower, |*S*_max_|, and the temperature at which it occurs, *T*_max_, in the bipolar regime: *E*_g_ = 2*e* × |*S*_max_| × *T*_max_. The estimated band gap amounts to *E*_g_ = 0.23 eV, in good agreement with the calculated gap, supporting the hypothesis that NaZnSb is a narrow gap semiconductor.

The total thermal conductivity can be deconvoluted into the sum of the electronic contribution and a lattice contribution, *κ_total_* = *κ_e_* + *κ_L_* = *L∙*T∙/ρ + *κ_L_*, where *κ_e_* is the electronic thermal conductivity, κ_L_ is the lattice thermal conductivity, and *L* is the Lorenz number. For metals and degenerate semiconductors with high carrier concentrations, the value of the Lorenz number approaches the Sommerfeld limit, *L* = 2.45 × 10^−^^8^ W∙Ω∙K^−2^, within the frame of the free electron model. The *L* estimated from the measured Seebeck coefficient data [28] yields similar values of 1.68 × 10^−^^8^ to 2.47 × 10^−^^8^ W∙Ω∙K^−2^. Thus, the Sommerfeld limit Lorentz number, *L* = 2.45 × 10^−^^8^ W∙Ω∙K^−2^ was used. The estimated electronic thermal conductivity is less than 2% of the total thermal conductivity, implying that the majority of the total thermal conductivity is due to lattice conduction. The room temperature lattice thermal conductivity is *κ_L_* = 1.5 W∙m^−1^∙K^−1^. Many Zintl antimonides have intrinsically low thermal conductivity, which was attributed to the structural complexity, and can be quantitatively related to the primitive unit cell volume, *V*, or number of atoms per unit cell, *N* [3,4,5]. The structure of NaZnSb is fairly simple with *N* = 6 atoms/unit cell and a unit cell volume of *V* = 146 Å^3^ (Table 1 and Table 2). The lattice thermal conductivity of 1.5 W∙m^−1^∙K^−1^ is on the lower side compared to the lattice thermal conductivity for other Zintl antimonies with similar structural complexity, such as layered SrZn_2_Sb_2_ [23] (*κ_L_* = 2.1 W∙m^−1^∙K^−1^ for *N* = 5 and *V* = 135 Å) or LiZnSb with a wurtzite structure [29] (*κ_L_* = 4 W∙m^−1^∙K^−1^ for *N* = 6 and *V* = 121 Å). The room-temperature lattice thermal conductivity, *κ_L_*, for NaZnSb is comparable to that for BaZn_2_Sb_2_ [30] (*κ_L_* = 1.6 W∙m^−1^∙K^−1^ for *N* = 20 and *V* = 552 Å). The latter has a more complex structure and a greater average atomic mass than that of NaZnSb. The low thermal conductivity of NaZnSb with a simple layered structure and light Na cation could be a result of stacking faults, similarly to the layered SrZnSb_2_ [3].

The thermoelectric figure of merit *zT* at room temperature amounts to 0.03, increases to 0.08 at 473 K, and finally reaches 0.23 at 675 K (Table 3). The experimentally determined *zT* values are in reasonable agreement with the predicted ones from the analysis of the band structures using the rigid band approach and semiclassic Boltzmann theory [31]. For the calculation of *zT*, the lattice thermal conductivity was fixed to *κ*_L_ = 2 W∙m^−1^∙K^−^^1^, which is higher than the experimentally determined one, *κ*_L_= 1.5 W∙m^−1^∙K^−1^. Using a similar calculation approach, LiZnSb (hexagonal wurzite structure type) was computationally predicted to exhibit remarkable thermoelectric efficiency with *zT* approaching 2 at 600 K [31], which was, however, not confirmed experimentally [29]. Our experimental study suggests that NaZnSb exhibits a heavily doped semiconductor behavior with a moderate thermoelectric efficiency, consistent with previously reported calculations.

## 4. Conclusions

NaZnSb was synthesized from the element and via hydride routes with only the hydride route allowing for the synthesis to be scaled up. Additionally, the hydride route results in the significantly shortened reaction time and improved the purity of the product. The thermal stability was studied through a synergistic combination of DSC, DSC-TG, and in situ high temperature synchrotron PXRD, which helped identify the limitations of each method in assessing the thermal stability of antimonides containing alkali metals. The thermoelectric properties of NaZnSb were experimentally measured, showing *p*-type narrow gap semiconducting behavior, and are consistent with the theoretical predictions. Our continued investigation of this and related systems using the facile hydride synthesis will allow for the compositional screening of new phases in the systems containing alkali metals.

## Figures and Tables

**Table 1:** Data collection and structure refinement parameters for NaZnSb.

Empirical Formula	NaZnSb
Space group, *Z*	*P*4/*nmm* (*No* 129), 2
Cell parameters: *a*, Å *c*, Å *V*, Å^3^	4.4201(6) 7.4776(11) 146.09(5)
Temperature, K	173(2)
Wavelenght, Å	0.71073 (Mo*K*_α_)
Absorption coefficient, mm^−1^	17.244
Range of *θ,* º	2.72 to 43.60°
Range of *h*, *k*, *l*	±8, ±8, ±14
Measured reflections	3039
Independent reflections	362 [*R*_int_ = 0.030]
Reflections with *I* > 2*σ*(*I*)	362; (*R*_σ_ = 0.046)
Data/restraints/parameters	362/0/10
Goodness-of-fit for *F^2^*	1.34
Final *R*-indices [*F* > 2*σ*(*F*)]	*R*_1_ = 0.024 *wR*_2_ = 0.024
*R*-indices (all data)	*R*_1_ = 0.049 *wR*_2_ = 0.049
Largest difference peak and hole, e/Å^3^	2.96 and −1.97

**Table 2:** Atomic coordinates and isotropic equivalent displacement parameters of NaZnSb.

Site	Wyckoff Site	*x*/*a*	*y*/*b*	*z*/*c*	*U*_eq,_ Å^2^
Na	2*c*	¼	¼	0.6518(3)	0.0120(4)
Zn	2*a*	¾	¼	0	0.0087(1)
Sb	2*c*	¼	¼	0.22187(3)	0.0064(1)

**Table 3:** Selected thermoelectric parameters of the NaZnSb compound.

*T*, K	*S*, μV∙K^−1^	*ρ*, Ω·m × 10^−4^	*κ*_total_ W∙m^−1^∙K^−1^	*zT*
This study, experimental				
303	193	3.0	1.48	0.03
473	208	2.6	1.31	0.08
675	244	1.1	1.16	0.23
Calculated*				
150				0.04–0.02 [31]
300				0.011–0.07 [31]
600				0.27/0.2 [31]

*zT values were calculated [31] using PBE-GGA and EV-GGA, respectively (PBE-GGA, Perdew–Burke–Ernzerhof generalized gradient approximations; EV-GGA, Engel–Vosko generalized gradient approximations). The lattice thermal conductivity was fixed to *κ_L_* = 2 W∙m^−1^∙K^−1^ and the relaxation time *τ* was set to 2 × 10^−14^ s.

## Supplementary Materials

The following are available online at http://www.mdpi.com/1996-1944/12/1/48/s1, cif file for NaZnSb; Figure S1, SEM images of SPS sintered NaZnSb pellet.
