# Supplementary material for: Thermal Stability and Thermoelectric Properties of NaZnSb
Source: Materials (Basel). 2018 Dec 24;12(1):48. doi: 10.3390/ma12010048 (PMC6337516; doi:10.3390/ma12010048)
Supplement: Supplementary file 1 [file materials-12-00048-s001.pdf]

materials

Article

Thermal stability and thermoelectric properties of NaZnSb

Volodymyr Gvozdetskyi [1], Bryan Owens-Baird [1,2], Sangki Hong[1] and Julia V. Zaikina [1,*]

[1] Department of Chemistry, Iowa State University, Ames, IA 50011, United States; volodya@iastate.edu (V.G.); skhong@iastate.edu (S.H.)

[2] Ames Laboratory, U.S. Department of Energy, Ames, IA 50011 United States; bowens@iastate.edu (B.O-B.)

* Correspondence: yzaikina@iastate.edu; Tel.: +1-515-294-3125

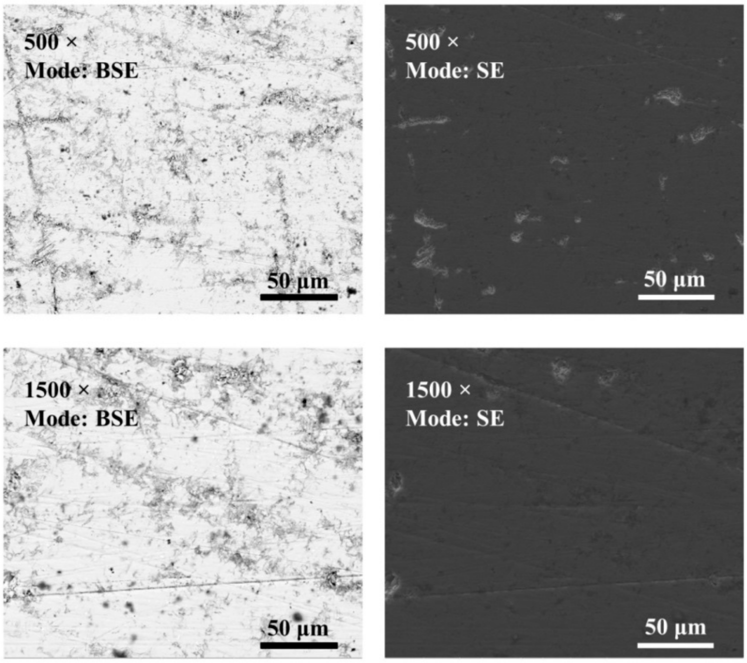

Figure S1. Scanning electron microcopy (SEM) images obtained in back-scattered electrons (BSE, left) and secondary electrons (SE, right) modes for the sample of NaZnSb prepared via hydride route and further densified by SPS. The composition determined by EDXS is $Na_{1.3(1)}Zn_{1.0(1)}Sb_{1.0(1)}$. The standard deviation in composition is calculated for the data collected in 8 different areas of each sample. The combined error of EDXS analysis is ± 5 %.
